# Resilience assessment in complex natural systems

**DOI:** 10.1098/rspb.2024.0089

**Published:** 2024-05-29

**Authors:** Camilla Sguotti, Paraskevas Vasilakopoulos, Evangelos Tzanatos, Romain Frelat

**Affiliations:** ^1^ Department of Biology, University of Padova, Padova 35100, Italy; ^2^ Institute of Marine Ecosystems and Fishery Science (IMF), Center for Earth System Research and Sustainability (CEN), University of Hamburg, Hamburg 22767, Germany; ^3^ European Commission, Joint Research Centre (JRC), Ispra, Italy; ^4^ Department of Biology, University of Patras, Patras 26504, Greece; ^5^ PO Box 30709, International Livestock Research Institute, Nairobi 00100, Kenya

**Keywords:** resilience, regime shifts, tipping points, climate change, stochastic cusp model, ecosystem-based management

## Abstract

Ecological resilience is the capability of an ecosystem to maintain the same structure and function and avoid crossing catastrophic tipping points (i.e. undergoing irreversible regime shifts). While fundamental for management, concrete ways to estimate and interpret resilience in real ecosystems are still lacking. Here, we develop an empirical approach to estimate resilience based on the stochastic *cusp* model derived from catastrophe theory. The *cusp* model models tipping points derived from a *cusp* bifurcation. We extend *cusp* in order to identify the presence of stable and unstable states in complex natural systems. Our *Cusp Resilience Assessment* (CUSPRA) has three characteristics: (i) it provides estimates on how likely a system is to cross a tipping point (in the form of a *cusp* bifurcation) characterized by hysteresis, (ii) it assesses resilience in relation to multiple external drivers and (iii) it produces straightforward results for ecosystem-based management. We validate our approach using simulated data and demonstrate its application using empirical time series of an Atlantic cod population and marine ecosystems in the North Sea and the Mediterranean Sea. We show that *Cusp Resilience Assessment* is a powerful method to empirically estimate resilience in support of a sustainable management of our constantly adapting ecosystems under global climate change.

## 1. Introduction

Ecological resilience, i.e. the capability of an ecosystem to maintain the same structure and function under the influence of external drivers, and avoid crossing catastrophic tipping points, is one of the emerging concepts of sustainability [1,2]. While maintaining ecosystems or populations resilient to global cumulative stressors is fundamental for ecosystem health (the condition of a system in which its attributes are in a normal range, i.e. it can provide a range of ecosystem services) [3], we are lacking concrete and simple ways to empirically estimate and interpret ecological resilience in real ecosystems [3–5]. This shortcoming is partly owing to inconsistencies related to the resilience concept (existence of different definitions across disciplines), but especially owing to the inherent complexity of natural systems [6–8]. The lack of solid methodologies to empirically estimate ecological resilience has caused its neglect in ecosystem-based management frameworks. For instance, European maritime policies (such as the European Union’s Marine Strategy Framework Directive (MSFD)) have only partially embraced the concept, even though the understanding of a system’s ecological resilience could facilitate the adoption of more adequate management measures [9–12].

Ecological resilience is embedded within the concept of regime shifts, i.e. nonlinear and abrupt transitions of a system between alternate states that differ in configuration and/or properties [4,13,14]. Indeed, a highly resilient system is able to withstand more pressures, staying clear from tipping points and associated regime shifts [14,15]. As a result, systems with low ecological resilience need to be managed with caution in order to avoid transitions to unwanted configurations [16]. By contrast, if a system is locked within an undesirable state (e.g. an overfished ecosystem), actions to erode its resilience would be needed to facilitate a shift towards a desirable state [4]. Knowledge about past resilience dynamics and regime shifts of ecosystems would help policymakers understand whether a system has undergone abrupt regime changes and identify the relevant external drivers [17–20]. Importantly, reliable empirical ecological resilience estimates are crucial for assessing the ability of a system to recover to previous baselines, often a key objective of ecosystem-based management, or for detecting if the new state is potentially irreversible [21,22].

Resilience-related terminology is still not so strictly defined, rendering its assessment more difficult. For example, in coral reef studies, ‘phase shifts’ are used to describe transitions between ‘alternative stable states’, which may be irreversible, while ‘alternative stable states’ are also used in the context of reversible shifts [23]. Moreover, resilience is a multifaceted concept that is still evolving over time. Besides ecological resilience, that is the focus of this study, resilience may also refer to how fast a system recovers from perturbation (engineering resilience) and whether a system is able to adapt and transform under new pressures (social resilience). Meanwhile, similar concepts like resistance or stability typically refer to a single (current) state, and they are more related to engineering resilience [15]. While measuring ecological and engineering resilience is relatively easy from experiments or theoretical models, the challenge lies in determining resilience from empirical data for large ecological systems that cannot be experimentally manipulated [8,24–26]. A number of methods have been proposed to estimate resilience in aquatic populations, communities and ecosystems [8]. Early warning signals of regime shifts estimate ecological and engineering resilience based on mathematical properties of time series representing the systems [3,4,27]. Other approaches to estimate engineering resilience in ecosystems are based on species interactions and make use of properties of network analysis and food-web modelling [28–30]. Other approaches quantify the ecological resilience of alternate states in probabilistic terms using information from a large number of different systems [31,32] or simulated system states [33], and/or fitting alternative models. Approaches based on model fitting/maximum likelihood can also be used to probe the resilience of complex natural systems [34,35]. A common issue across all these methods is that they generally require large and detailed (empirical or simulated) datasets with high temporal resolution, which are usually unavailable for complex natural systems.

An empirical approach for the detection of regime shifts and the quantification of ecological resilience that makes use of typical ecological monitoring time series is the Integrated Resilience Assessment (IRA) [27,36]. IRA quantifies resilience by identifying two or more multiple stable states of a system, affected by one driver that acts as a system stressor. The system states are identified by testing the fit of multiple models on the response curve of the system to a stressor. If a discontinuous response is identified, the predicted lines of the optimal model represent the attractors and form the basis to estimate the position of the tipping points. Ecological resilience is then estimated based on how far each system state lies from both its attractor and its respective tipping point [27,36–39]. This method has been applied across different marine ecosystems [27,36,38,40,41]. The IRA, has however, the shortcoming that it is a single-driver approach and hence a strong simplification of complex ecological systems that are usually affected by multiple and possibly interacting drivers [42]. Moreover, the underlying statistical model does not represent potential irreversibility [42]. Models based on the catastrophe theory such as the stochastic *cusp* model (*cusp*) can better represent irreversible discontinuous dynamics driven by multiple drivers [8,43–46]. *Cusp* is a nonlinear modelling approach based on a cubic differential equation that allows to identify tipping points derived from a bifurcation in a system depending on two interactive drivers and to test for reversibility of system states [44,45,47,48]. The *cusp* model started to be used in the early 1970s in different disciplines (such as economics, ecology and behavioural science) but was soon criticized owing to its deterministic framework and the lack of stochasticity [49,50]. The recent addition of the stochastic framework has revived interest in this model that can describe discontinuous dynamics of a state variable around a bifurcation. Expanding *cusp* to estimate ecological resilience could be a step forward in developing a reliable resilience assessment from empirical data.

Here, we combine the *cusp* and concepts from IRA’s resilience estimation to develop a *Cusp Resilience Assessment* (*CUSPRA*) that has three characteristics: (i) it provides estimates on how likely a system is to cross a tipping point characterized by hysteresis (i.e. ecological resilience), (ii) it assesses resilience in relation to multiple external drivers and (iii) it produces straightforward results for ecosystem-based management. These three characteristics are fundamental for the development of a method that is both easy to apply on typical empirical datasets and facilitates a better understanding of how systems respond to external drivers. We validate CUSPRA using artificial data and demonstrate its application using empirical time-series data related to an Atlantic cod population and ecosystem dynamics in the North Sea and the Mediterranean Sea. We show that CUSPRA provides an advanced methodology to empirically estimate ecological resilience in support of a sustainable management of constantly changing and adapting ecosystems under global climate change.

## 2. Material and methods

### (a) Building the resilience indicator

**Figure 1.**
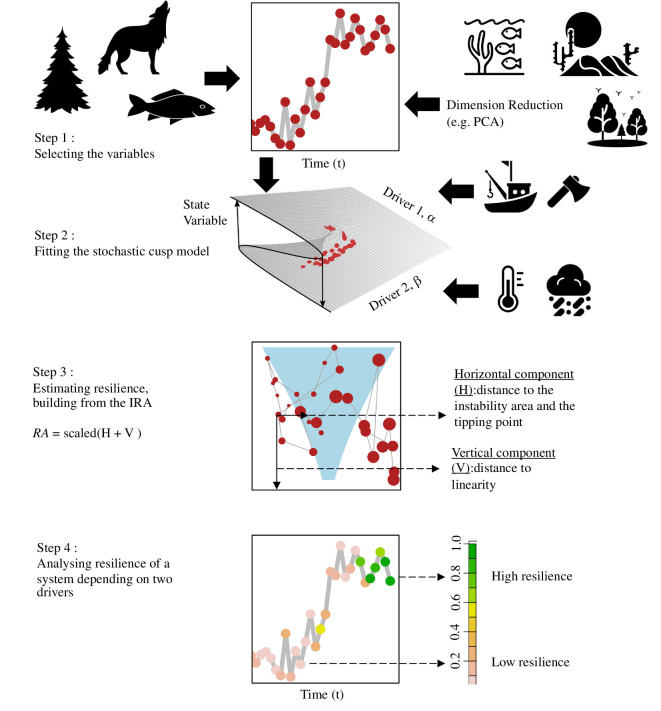
The four steps of CUSPRA. (i) Selecting the variables: the state variable should be a time series that can represent anything from a single population to an ecosystem. If multivariate time series are available, these need to be reduced to an ecosystem metric using a dimension reduction technique such as Principal Component Analysis (PCA). (ii) Fitting the stochastic *cusp* model: the model, using a cubic differential equation, represents the dynamics of a system from linear and continuous to nonlinear and discontinuous, depending on the combination of the two drivers. (iii) Estimating resilience (RA): projecting the results of the *cusp* model in two dimensions, an estimate of resilience can be calculated for every state in time depending on the two drivers. Resilience is calculated based on the vertical distance of a point to linearity and on the horizontal distance of a point to the *cusp* area (in light blue) or tipping point. . In the *cusp* area, three equilibria, two stable and one unstable, are possible, in the area below the fold. (iv) Analysing resilience depending on the two interactive drivers: CUSPRA allows to understand how resilience changes over time and to compare the results between case study systems.

We built CUSPRA following a four-step approach: (i) select the variables to use in the model, (ii) fit and evaluate the *cusp* model, (iii) estimate resilience and (iv) assess the results in relation to the interaction of two drivers (figure 1).

#### (i) Steps 1 and 2: Fitting the stochastic cusp model

The first two steps deliver a *cusp* model as the basis for the resilience estimation. The *cusp* represents discontinuous changes in a dynamic system depending on two control drivers (electronic supplementary material, figure S1) [47,51]. The system (i.e. a state variable) is represented by a vector of data, typically a time series, and can be anything from abundance estimates of a population to proxies of ecosystem state(s), depending on the system whose resilience we are interested in analysing. The dynamics of the state variable depending on two interactive drivers are modelled with a cubic differential equation extended with a Wiener’s process to add stochasticity (equation (2.1)) [43,44,46]. The rate of change of the system depends on two parameters or factors, *α* and *β*.


(2.1) $$\frac{-\delta V\left(z,\alpha ,\beta \right)}{\delta z}=\left(-z_{t}^{3}+\beta z_{t}+\alpha \right)dt+\sigma_{z}dW_{t},$$

*α* and *β* are the two factors whose interaction create a bifurcation for the state variable. *α* is the asymmetry factor and controls the state of the system, thus inducing system changes, for instance from regime A to regime B. This factor is usually a linear model of one or multiple external drivers that control the dimension of the state variable such as fishing effort in harvested ecosystem systems [47,52,53] (equation (2.2)). A second bifurcation factor *β* modifies the relationship between the asymmetry factor and the system state from linear continuous to nonlinear and discontinuous and thus shapes the functional form of system dynamics, i.e. if the system approaches the bifurcation. The bifurcation factor is usually a linear model of one or more external drivers that induce changes in the system, such as temperature, eutrophication or climate indices [47,52–54] (equation (2.2)). Abiotic and biotic drivers and their combination can be used to model the three factors fitted in the stochastic *cusp* model.


(2.2) $$\left\{ \begin{array}{l} \alpha =\alpha_{0}+\alpha_{1}Fishing\\ \beta =\beta_{0}+\beta_{1}SST\\ z_{t}=w_{0}+w_{1}Population, \end{array}\right.$$

where *α*_0_, *β*_0_ and *w*_0_ are the intercepts, and *α*_1_, *β*_1_ and *w*_1_ the slopes of the models. SST stands for Sea Surface Temperature.

The choice of which variable represents the bifurcation and the asymmetry factor can be made in different ways. If there is a deep knowledge of the system, the choice can be made ‘*a priori*’. For instance, in the case of a harvested stock, it is reasonable to believe that fishing is the driver that has the biggest impact on the stock and thus can lead to a shift of the stock from state A to state B, while temperature might have a more indirect effect on the stock by changing its productivity. Thus, in this case, fishing will be fitted as the asymmetry factor and temperature as the bifurcation factor. In other examples, where there is not such a deep knowledge of the systems or the mechanisms of the state variable might be unknown, a model selection based on the Akaike Information Criterion (AIC) could be performed to understand which combination of variables best describes changes in the state variable. In any case, it should be noted that the stochastic *cusp* model provides a method to prove and describe how the bifurcation may have occurred but does not provide a mechanistic explanation of the phenomenon.

The solution of the cubic differential equation of the model can either detect the multiple equilibria in the state variable, indicating discontinuous dynamics and a regime shift, or identify that just one equilibrium exists, and thus linear continuous dynamics prevail [44,47,48,53]. The model is generally represented in a horizontal plain determined by the combination of the two drivers (figure 1, steps 2 and 3). The system can move in the plain from areas in which just one equilibrium exists, i.e. linear dynamics, to areas with multiple equilibria, i.e. discontinuous dynamics (i.e. a regime shift, the light blue area) (electronic supplementary material, figure S1). This area is also identified as the area below the fold, where tipping points can occur and resilience is low (electronic supplementary material, figure S1) [44,46]. To detect the presence of discontinuous dynamics, Cardan’s discriminant is calculated (equation (2.3)).


(2.3) $$\delta =27\alpha^{2}-4\beta^{3}$$

If the Cardan discriminant is smaller than or equal to 0, then the state variable follows nonlinear discontinuous dynamics and has multiple equilibria, while if it is higher than 0 the system will follow continuous dynamics [46].

Apart from Cardan’s discriminant, the stochastic *cusp* model directly fits two alternate models, one linear (equation (2.4)) and one logistic (equation (2.5)) in order to select between different dynamics of the system.


(2.4) $$Z_{t}=g_{0}+g_{1}\cdot Fishing+g_{2}\cdot SST+\epsilon ,$$

where *g*_0_ is the intercept of the model, *g*_1_ and *g*_2_ are the slope coefficients of the two control variables and ϵ is the normally distributed random error (mean = 0, variance= *σ* square).

The logistic model, showing nonlinear but continuous dynamics is fitted as:


(2.5) $$Z_{t}=\frac{1}{1+e^{\left(\frac{-\alpha }{\beta^{2}}\right)}}+\epsilon ,$$

where *Z*, *α* and *β* are canonical variables of the observed state and control variables defined in equation (2.2) and ϵ is the 0 mean random disturbance.

Before proceeding with the resilience estimation, it is necessary to evaluate the model and check that the stochastic *cusp* model is the better model compared with the alternative linear model, since the definition of ecological resilience includes the presence of alternative stable states and irreversible tipping points. We recommend using four criteria to establish whether the *cusp* is a good model to describe the data [46,47].

— The pseudo *R*-squared is higher than 0.3. The pseudo *R*-squared is calculated as 1 − (error variance/var(*z*)), where the error variance is defined as the difference between the observed values and the mode of the distribution closest to these values.
— The corrected Akaike Information Criterion (AICc) of the *cusp* model is lower than the AICc of the linear model (by at least 2 units).
— The percentage of points inside the *cusp* area needs to be more than 10%.
— The estimate of the state variable parameter needs to be significant.

If these four criteria are met, the stochastic *cusp* model is superior to the alternative model and is a good simplification of the state variable dynamics; thus, it is possible to proceed with the resilience estimation.

Before proceeding, we tested if the four criteria were able to correctly validate the use of the *cusp* model. To do this we applied the stochastic *cusp* model to 100 simulated chaotic, random, periodic and *‘cusp*’ time series. The periodic time series were generated with a period of 10 time steps and little noise (standard deviation, s.d. = 0.3), the random time series with a mean = 0 and s.d. = 1. The chaotic time series were generated following a logistic map. We fixed the control parameter (*r*) at 3.9 in order to explore similar types of a chaotic system. Finally, the *α* and *β* used in the models were simulated as *α* being strongly autocorrelated (AR1 = 0.9) and *β* being normally distributed. The mean of *β* varied in five different model scenarios, from −2 (mostly linear) to 2 (mostly discontinuous) with an increment of 1. We ran 10 repetitions of each scenario with a time series of 50 time steps. The state variable for the *‘cusp*’ time series was then generated from *α* and *β* (electronic supplementary material, figure S2). The simulations can be found at: https://github.com/rfrelat/cuspra/tree/master/inst/scripts/simCUSPRA.R.

#### (ii) Step 3: estimating resilience, building from the IRA

Ecological resilience is estimated similarly to the IRA, by calculating the distance to the instability area determined by the combination of the two control variables (*α*, *β*), and using two components, a vertical and a horizontal component [27]. The resilience of the state variable *z* is high when the combination of the observed control variables (*α*, *β*) that define *z* are far from the bifurcation area while the resilience is low when *α*, *β* are within the bifurcation set. Hence, the resilience of the state variable *z*, which is appropriately modelled by a *cusp* and defined by two control variables (*α*, *β*) depends only on the values of (*α*, *β*) and is independent of the value of *z*. By definition from equation (2.3), the bifurcation set is defined by equation (2.6):


(2.6) $$\left\{ \begin{array}{l} \alpha_{bif}=2*{\left(\frac{\beta }{3}\right)}^{\frac{3}{2}}\;\;\;\;if\beta >0\\ \alpha_{bif}=0\;\;\;\;\;if\beta <0, \end{array}\right.$$

The horizontal component of resilience is the distance of α from the bifurcation set or instability area equation (2.7).


(2.7) $$H=abs\left|\left(\alpha \right)\right|-\alpha_{bif}.$$

*H* is negative when inside the bifurcation set (abs(*α*)< $\alpha_{bif}$ , low resilience) and positive when outside the bifurcation set (higher resilience).

The vertical component is the distance to linearity, defined by the bifurcation variable β , representing how discontinuous the system is (equation (2.8)).


(2.8) V=−β.

*V* is negative when discontinuous and positive when linear (*β* < 0).

The resilience (*Ri*) is then the weighted average between the horizontal and the vertical components. We give double weight to the horizontal component (*H*) to further stress the importance of α for the resilience of the system in comparison to the vertical component (equation (2.9)).


(2.9) $$Ri=\frac{2H+V}{3}.$$

Highly negative *Ri* indicates a highly discontinuous system in the bifurcation set, while highly positive values indicate a linear system far from the bifurcation set. We transformed this resilience value (*Ri*) using hyperbolic tangent transformation to get an indicator of resilience (*RA*) between 0 and 1, with 0 reflecting low resilience and 1 high resilience (equation (2.10)).


(2.10) $$RA=\frac{tanh\left(Ri\right)+1}{2},$$

**Figure 2.**
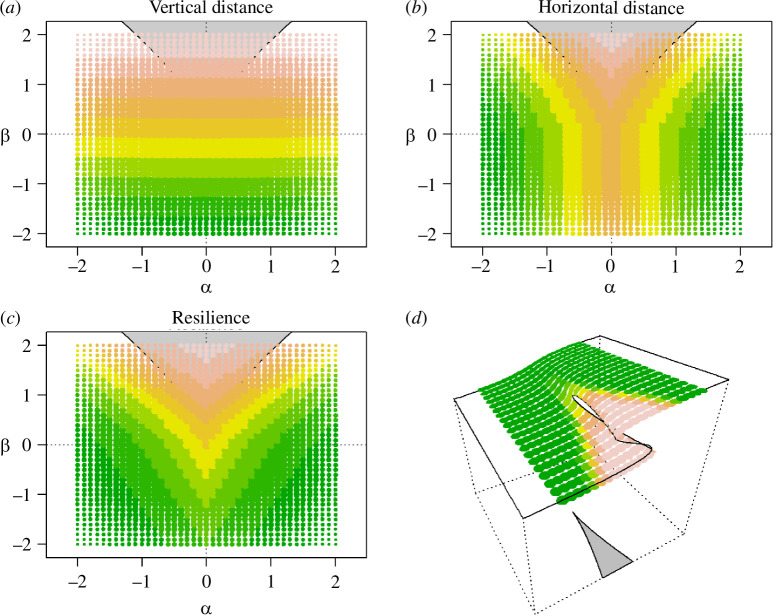
The CUSPRA resilience indicator (*RA*). The two-dimensional representation of the stochastic *cusp* model; for *a*–*c*: on the *x-*axis, *α*, the driver controlling the dimension of the state variable; on the *y*-axis, *β*, the driver controlling whether the relationship between *α* and the state variable is linear and continuous or nonlinear and discontinuous. In grey, the instability area is determined by the combination of *α* and *β* values where three equilibria are possible. The colours of the areas of the two-dimensional panel, correspond to different resilience estimates (pink = 0 and green = 1). (*a*) The vertical component of resilience where resilience is estimated as the distance from linearity. (*b*) The horizontal component of resilience is estimated as the horizontal distance from the *cusp* area. (*c*) The CUSPRA resilience indicator is obtained by summing the vertical and horizontal resilience estimates. The resilience is lower inside the instability area. (*d*) The CUSPRA resilience values and the three-dimensional landscape represent the *cusp* sudden bifurcation.

In this way, we obtained an indicator that can be comparable across multiple systems and multiple populations. The resilience estimation is computed for every point of the state variable, depending on the two control variables. Thus, if we fit as a state variable the time series of the biomass of a population, for every point in time, a value of *RA* will be calculated. A low *RA* (value close to 0) means that with regard to the fluctuation levels of the stressors, the system is unstable, i.e. large changes in state can happen with little changes in the stressor variables and that the system is close to a tipping point (figure 2). On the contrary, a large *RA* (close to 1) indicates that the system is mostly linear, and thus if the control variables change the system will change linearly (i.e. the system is far from the tipping point) (figure 2). Being based on the *cusp* our concept of resilience does not test for resilience *per se* (as a ‘condition’ of the system independently of the variables that may affect it) but estimates the resilience depending on two external drivers and determines how close a system, which presents a *cusp* bifurcation, lies to a tipping point.

#### (iii) Step 4: testing the indicator

To test whether the indicator provides meaningful results, we created state variables from multiple stochastic *cusp* models having the *α* variable being strongly autocorrelated (AR1 = 0.9) and the *β* variable being normally distributed. The mean of *β* varied in five different model scenarios, from −2 (mostly linear) to 2 (mostly discontinuous) with an increment of 1. We ran 10 repetitions of each scenario with a time series of 50 time steps.

We tested three hypotheses to validate our resilience metric:

**Hypothesis 1 (H1)**: the resilience decreases with the level of nonlinearity (*β*).

**Hypothesis 2 (H2)**: a state with low resilience has a higher probability of large changes.

**Hypothesis 3 (H3)**: a state with low resilience is linked to anomalously large variance.

H1 helped us to show how the model works, while H2 and H3 validate that our concept of resilience corresponds to the one described in the literature, where a low resilient system exhibits higher variance and higher probability to change [45,55].

To test these hypotheses, we estimated the CUSPRA resilience on each simulation. For H1, we compared the minimum *RA* to the set level of nonlinearity (*β*). We expected that the minimum *RA* decreases with nonlinearity so it should increase with the mean level of *β*. For H2, we compared the absolute difference between successive state values with the value of *RA*. We expected that absolute differences would decrease with higher *RA*. For H3, we computed the variance within a five-time step window, and we compared it to the level of *RA*. We expected that the variance is low for high resilience, and that the variance increases when resilience decreases.

### (b) Data

To test the newly developed indicator, we used data from four published scientific articles using the stochastic *cusp* model or the IRA and ranging from populations to community and to trait configurations [37,38,48]. We built an R package (cuspra) and a Shiny App to allow all members of the scientific community to test our resilience indicator with their own data (https://rfrelat.shinyapps.io/CUSPRA/). In all three examples, the drivers tested to assess resilience were fishing and temperature (i.e. Sea Surface Temperature (SST)), derived from different sources. The examples are thus comparable. To test population resilience, we used data from the Atlantic cod (*Gadus morhua*) stock of the northeast Arctic Sea [48]. We used as a proxy for the state of Atlantic cod in the northeast Arctic, Spawning Stock Biomass (SSB) data deriving from stock assessment. Fishing mortality (F), also estimated from stock assessment, and SST were used as drivers to test resilience. The data ranged from 1946 to 2016 [48] (electronic supplementary material, figure S3).

Community resilience was explored using North Sea and Mediterranean Sea data from previous studies. In this case, to build a ‘community state’, first a multivariate reduction technique (PCA) needed to be applied [37]. CUSPRA, similarly to both IRA and the stochastic *cusp* model, needs to fit a single vector (a time series) representing the system state to the model. The North Sea community state index was built by performing a PCA to a matrix of data coming from the Continuous Plankton Recorder (CPR) and International Bottom Trawl Survey (IBTS) [53]. SST derived from NOAA ErSST v5 and fishing effort (expressed as hours swept per year) from Couce *et al*. (2019), were used as drivers to test resilience [56,57]. The data ranged from 1985 to 2019 (electronic supplementary material, figure S4) [53]. In the Mediterranean case, a community state was built using landing data extracted from the database of the Food and Agriculture Organization (FAO) of the United Nations and then reduced using a PCA [37]. As drivers, fishing capacity (i.e. cumulative fleet gross tonnage) estimated for the entire EU Mediterranean fleet (EU fleet capacity/gross tonnage) and SST from the NASA PODAAC were used. The data ranged from 1985 until 2013 (electronic supplementary material, figure S5). Finally, to estimate resilience of the biological traits of the Mediterranean Sea community, we used landing traits data [37]. This dataset was derived by combining the Mediterranean fisheries landing data of the FAO for the years 1985–2015 (https://www.fao.org/fishery/en/collection/gfcm_capture?lang=en) with a matrix including data on 23 complete traits related to the biology, ecology, trophic role, distribution, habitat and behaviour of 205 species (mostly fish, but also molluscs and crustaceans) [58]. The multiplication of the two datasets resulted in a matrix of community-weighted mean trait landings by year in the Mediterranean Sea [37] (electronic supplementary material, figure S6).

All analyses were performed in R (version 4.0.2) using the package *‘cusp’* [47] for the *cusp* modelling. The implementation of the calculation of the resilience indicator (*RA*) is documented in an R package available in the github repository (https://github.com/rfrelat/Cuspra) and can be downloaded directly in R by typing: devtools::install_github (‘rfrelat/cuspra’). A Shiny app was created and made available to help scientists calculate the resilience indicator on their time series or run other simulations (https://rfrelat.shinyapps.io/CUSPRA/). The data and codes are additionally stored in Dryad [59] and in Zenodo [60].

## 3. Results

### (a) Building the resilience indicator

We have created a protocol to effectively apply CUSPRA (figure 1). The protocol starts with the definition of the system for which the ecological resilience will be estimated as well as the relevant pressures. The system may be described by a single variable (e.g. biomass representing a fish stock) or multiple variables (e.g. abundance of multiple species representing a community). For the latter, applying a reduction technique such as PCA is needed to summarize the multidimensional system into a single variable, so that the stochastic *cusp* model can be fitted. The model fit must be evaluated before proceeding to the last step, the resilience estimation. The framework, starting from a time series of a state variable and two drivers, provides an estimate of the resilience of the state variable for each combination of the drivers.

### (b) Validation

First, we validated the use of the four criteria to correctly classify a time series exhibiting a catastrophic shift. Our results show that the four criteria together have a high power of correctly rejecting the application of a stochastic *cusp* model to a time series without a *cusp* bifurcation dynamic. Indeed, there were almost no false positives (1%) and some false negatives (8%). The false positive mostly involved rejecting *cusp* simulations when there is a low number of points in the *cusp* areas. The pseudo-R-squared criterion was best at rejecting the application of the stochastic *cusp* model to random time series, while the delta AICc was best with chaotic and periodic time series (electronic supplementary material, figure S7).

**Figure 3.**
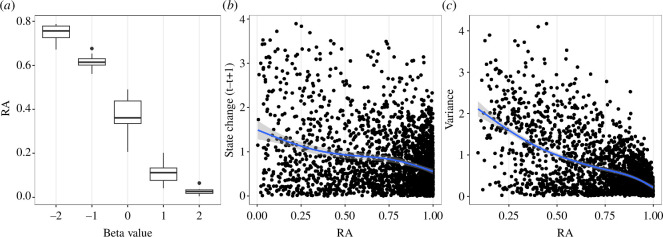
Simulation results for model evaluation. (*a*) Distribution of the minimum RA resilience (*y*-axis) for a simulated time series with different mean values of the *β* drivers (*x*-axis). (*b*) Changes in the state variable depending on its resilience (*x*-axis). The blue line represents the smoothed relationship between these two variables, showing that at lower *RA* values the probability of change increases. (*c*) Variance of the state variable depending on its resilience value *RA* calculated with a 5-year moving window. The blue line represents the smoothed relationship showing that variance decreases with increasing resilience.

Then, we validated our CUSPRA method by simulating time series of two drivers, *α* and *β*, to derive a state variable from the stochastic *cusp* model. We assumed the *α* variable to be strongly autocorrelated (AR1 = 0.9) and the *β* variable to be normally distributed, to imitate, respectively, an anthropogenic driver such as fishing, and a climate variable such as temperature or productivity. The mean of *β* varied in five different model scenarios, from −2 (mostly linear) to 2 (mostly discontinuous). We ran 10 repetitions of each scenario with a time series of 50 time steps. With these simulations, we tested three hypotheses to evaluate whether our model was meaningful. First, we wanted to show that resilience decreases with the level of nonlinearity (*β*). A *β* of – 2 has high resilience while a *β* of 2 has a resilience close to 0 indicating that the choice of a meaningful bifurcation variable to estimate resilience in real examples is crucial (figure 3*a*). The second and third hypotheses were related to the concept of resilience itself, i.e. a system with low *RA* has a higher probability of change and is linked to larger variances. These two properties are related to the concept of critical slowing down, a property typical of systems approaching a tipping point [28,61]. Using our simulated data, we confirmed that the amount of change of the state variable between two time points *t* and *t*_+1_ (delta Δ) increases and becomes more variable as *RA* declines (figure 3*b*). Moreover, at a low *RA* the variance of the state variable (calculated with a 5-year moving window) increased (figure 3*c*). These results indicate that the resilience estimated by CUSPRA corresponds to the concept of resilience in ecology [4,46,62,63].

### (c) Application to real systems

**Figure 4.**
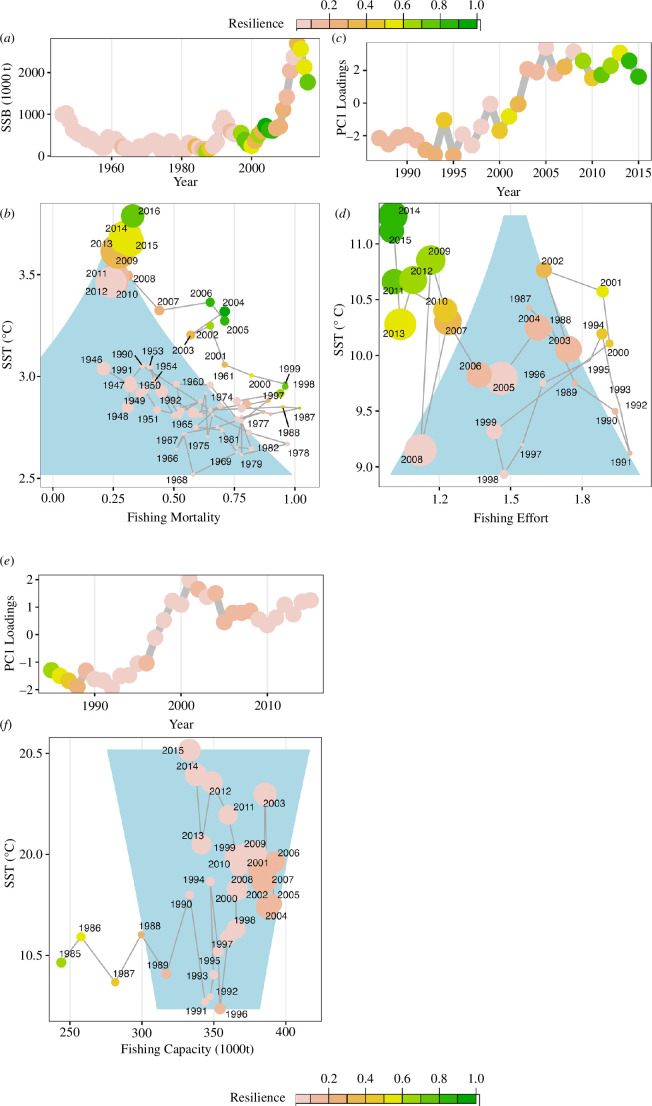
The CUSPRA application. (*a*,*b*) Resilience of northeast Arctic cod spawning stock biomass (SSB) depending on fishing mortality and Sea Surface Temperature (SST). (*c*,*d*) Resilience of the North Sea ecosystem depending on fishing effort and SST. (*e*,*f*) Resilience of the Mediterranean ecosystem based on traits depending on fishing capacity and SST. *a*,*c*,*e* show the time series of resilience in the different systems. *b*,*d*,*f* show the resilience values in the two-dimensional representation of the *cusp* model with the blue area corresponding to the *cusp* area where three equilibria are possible, i.e. two stable and one unstable. The dimension of the dots is proportional to the magnitude of the state variable. The colour of the dots corresponds to the colour of our CUSPRA resilience estimate RA, pink = 0, low resilience, green = 1, high resilience.

We used four examples to illustrate the application of our new resilience estimation approach. First, we estimated resilience of a commercially important fish stock (Atlantic cod, *Gadus morhua*) in the northeast Arctic [48] based on fish stock assessment data (modelled population outputs). We used SSB (the weight of mature fish in the stock) as the predictor for the state variable, while α was predicted by fishing mortality (F), and *β* by SST (for more details about the modelling see Sguotti *et al.,* 2019, electronic supplementary material, figure S2). The *cusp* model fitted well and was superior to the alternative linear model (electronic supplementary material, table S1). All four validation criteria were met and thus the *cusp* model fitted well to the data. The analysis shows that at the beginning of the time series the resilience of the stock was low (figure 4*a*,*b*). During that period the stock biomass was low, fishing mortality on a medium level and temperature low as well. The subsequent increase in fishing pressure pushed the stock towards a resilient and hence stable (albeit undesirable) low biomass state, outside the *cusp* area (figure 4*a*,*b* green, small dots). Decreasing fishing mortalities after 2007 coupled with an increase in temperature eroded the resilience of the biomass (pink dots) and moved the system back into the *cusp* area, leading to a tipping point from a low towards a high biomass state in *ca* 2009. A further increase in temperature subsequently increased the resilience of the high biomass state (figure 4*a*,*b*). The northeast Arctic cod provides an example in which a management measure (i.e. reduced fishing pressure) eroded the resilience of the unfavourable low biomass state, and in combination with increasing temperatures moved the stock towards a more favourable stable state, representing a positive transition.

Next, we applied CUSPRA to the dynamics of entire communities (North Sea and eastern Mediterranean Sea) represented by multivariate datasets. We used as a state variable the first mode of variability derived from the PCA (PC1). We predicted *α* with a time series of fishing pressure (see §2) and *β* with SST. The *cusp* models of the North Sea fitted well and passed the four criteria (electronic supplementary material, table S1). The analyses revealed very low resilience in the North Sea community at the beginning of the time series (figure 4*c*,*d*) when fishing pressure was high and temperature low, and the community was in a state dominated by cod [53]. With decreasing fishing pressure (mainly in the 2000s) and increasing temperature (mainly in the 2010s) the North Sea community changed from low to high resilience owing to structural changes in terms of species composition [53]. On the contrary, the eastern Mediterranean Sea community model did not pass the four criteria. Indeed, the AICc of the linear model was lower than the AICc of the *cusp*. Therefore, this community presents another type of dynamic that cannot be modelled with CUSPRA.

Finally, we used CUSPRA to estimate resilience of the trait composition of the fish community of the Mediterranean Sea [38]. PC1 here represents the main mode of variability of the trait space of the fish community and was used to predict the state variable in the *cusp* model. Again, we used a measure of fishing pressure (EU fleet capacity/gross tonnage) and SST to predict the *α* and *β* variables, respectively (electronic supplementary material, figure S5). The *cusp* model fitted well and met all four criteria (electronic supplementary material, table S1). Similar to the analysis of the eastern Mediterranean fish community, resilience decreased initially with increasing fishing pressure (figure 4*e*,*f*). With the decline of fishing capacity and especially the increase in temperature, the system abruptly shifted to a new state (see also Tsimara *et al*. 2021) in the late 1990s, being however, of low resilience, i.e. prone to further shifts. The analysis denotes that only a drastic reduction of fishing pressure would be able to drive the system towards a resilient state, and the increase of SST will require more drastic management approaches.

## 4. Discussion

CUSPRA is a new method to assess the resilience of ecological systems based on empirical data. This method provides an estimate of how close a system is to a tipping point (in the form of a *cusp* bifurcation) and hence to a shift into a new state or regime. Such dynamics are represented in CUSPRA through the application of the stochastic *cusp* model [64], a mathematical approach that can detect bifurcation in a system and thus identify tipping points [9,46,48,54]. Moreover, CUSPRA estimates resilience based on the effect of multiple interacting drivers and provides an indicator directly applicable to ecosystem-based management. We validated our approach using simulated data and tested our newly developed resilience indicator using four empirical example datasets comprising three different system types, i.e. a fish population, two fish communities and a community of traits [37,38,48]. Interestingly, by using the same drivers in all case studies, we can understand their varying impacts in the different systems. For instance, the increase in temperature in the North Sea ecosystem, coupled with strong management measures (i.e. decreased fishing pressure) [53,65], has led to a new resilient ecosystem state, while in the Mediterranean Sea (in the community example described by traits) similar changes in temperature but an increasing exploitation caused a new state but with low resilience. In the Mediterranean case, fishing pressure appears to be still excessively high and the system is likely closer to its maximum thermal tolerance [66,67]. Our examples demonstrate that CUSPRA is useful to understand the resilience of a system and how close it is to tipping points. Our new method not only advances empirical studies in resilience science but can also be directly applicable in ecosystem management settings, even beyond the marine environment, since it is easily transferable to a variety of systems, not only ecological systems but also socio-ecological, financial or behavioural systems.

Another characteristic of our approach is that resilience is quantified based on the interaction of multiple stressors and thus it quantifies hysteresis to interactive pressures [46,48]. This is a step forward compared with other methods that estimate resilience without accounting for system drivers, or methods such as the IRA, that estimate resilience depending on only one driver [28,36]. In the Anthropocene, multiple drivers acting in an interacting or cumulative fashion are increasingly likely to impact our resources and ecosystems, so methods that can consider a higher level of complexity are more suitable to model real systems [68,69]. It is possible to fit in the CUSPRA even more than two drivers adding multiple variables to the two factors controlling the system states. These would allow for a multidimensional study of resilience [70]. Finally, the estimation of a resilience indicator ranging from 0 to 1 allows the CUSPRA to be easily translatable into management and to be used for comparisons across multiple systems. The simplicity of the final indicator is appealing for management purposes since it constitutes a straightforward, unitless metric indicating whether the system under management is in a state that is rather stable or prone to change. Moreover, the possibility of comparing and understanding the resilience of different populations, ecosystems or socio-ecological systems to the same stressors can improve our knowledge about resilience and favour a better comprehension of this complex concept. Thus, our new method can translate the multifaceted concept of resilience in an easily comprehensible metric that can be used in many different disciplines providing useful information for management purposes. Examples of how to translate the concept into management can be found in electronic supplementary material, table S2.

Our CUSPRA approach takes the quantitative resilience assessment of complex natural systems based on empirical data a step forward. While our new method better represents resilience based on the effect of two interacting drivers, it also shows some limitations linked with the stochastic *cusp* model regarding the consideration of autocorrelation in the time series of the variables and concerning model evaluation [47,48]. Several indicators hence need to be evaluated before a *cusp* model can be assessed to be superior to alternative linear and continuous models [47,48], i.e. before CUSPRA can be used to assess resilience. Performing a simulation, we showed that the four criteria used to evaluate the *cusp* model successfully manage to discriminate true *cusp* bifurcation dynamics. In all three out of four case examples, the cusp model was found to provide more reliable fits to the data than alternative models, which indicates that these ecological systems exhibited nonlinear and discontinuous dynamics.

The model also presents opportunities for improvement. At present, our approach is ‘data-driven’, and thus it is difficult to make predictions. Indeed, the method can be used to estimate resilience of a system in hindsight only, while it cannot provide inferences about its future development [48,54]. Nevertheless, inspecting the interactive effects of drivers and the position of the state variable relative to the instability area provides information on the likely development in the near future. Other future developments could be to extend CUSPRA to the possibility of detecting more than two stable states. This limitation resides in the *cusp* model that is only able to depict two alternative states and hence no multiple consecutive tipping points can be assessed. Other bifurcation models, such as the ‘butterfly’, should be able to detect more than two stable states and thus could also be integrated in CUSPRA [46]. Nevertheless, usually empirical time series are short, and it is reasonable to believe that only two states of the system likely exist in the data time scales. If longer time series are to be fitted to the data, an extensive data analysis before fitting the CUSPRA is necessary. Visualizing the development of the state variable will show if fitting two separate CUSPRA models is necessary [71]. Other types of bifurcation which resemble the *cusp* (e.g. Hopf, Neimark-Sacker) might arise in natural systems. At present, our model and indicator might have difficulties in discriminating these bifurcations. However, CUSPRA is the first developed indicator that estimates resilience from bifurcations in empirical systems, and therefore, it is already an important step forward in ecology. CUSPRA models very complex phenomena in a simplistic way [9]. While this simplification could be criticized, it is one of the strengths of this approach, as it makes CUSPRA outputs easily translatable into management. Nonetheless, it would be beneficial for a CUSPRA analysis to be supported by additional indicators aiming to better understand the system state.

### (a) How could CUSPRA be used in ecosystem-based management settings?

First, knowledge on the type of dynamics that the system has exhibited in the past (linear or discontinuous) is important to understand if a system is vulnerable to regime shifts [10]. Knowing which drivers have caused the discontinuous dynamics, can give an indication on the resilience of the system towards these drivers, and thus how vulnerable the system is to them [5,72]. This is important for management since it can help to establish which drivers need to be managed in a more precautionary approach in order to enhance the resilience of the system and avoid the crossing of a tipping point [73]. Moreover, CUSPRA can determine the levels of the stressors at which the system will switch into a new state, i.e. the position of the tipping point. This is important knowledge for managers since it can help to mitigate stressors in order to avoid their critical levels [5,74]. Finally, the method gives a snapshot of the resilience of the system at present and thus can indicate whether a system will approach a tipping point in the immediate future. This is particularly important information in order to decide whether to adopt precautionary approaches or whether to try to restore the system towards initial conditions [5]. More precisely, if CUSPRA estimates low ecological resilience for a system, then management could apply a precautionary approach in order to avoid the tipping point. Instead, if a system shows high resilience, restoration towards a previous baseline might be impossible and management should be more focused on aiming for a sustainable transformation and maintaining the new status of the system. Potentially, applying CUSPRA using multiple drivers and their combinations could help managers to define a safe operating space of the system and thus favour better management approaches [75,76]. Estimating resilience is fundamental to properly managing natural systems; however, this concept is seldomly included in management owing to methodological limitations [2,3]. By estimating resilience of ecological systems impacted by multiple stressors, CUSPRA allows for a better quantification of resilience and a direct application to management which is urgently needed if we want to manage constantly changing and adapting systems under global climate change [23,77].

## Data Availability

The data are stored in Dryad [59]. The codes are available in Zenodo [60]. The package to perform the model can be downloaded directly in R by typing: devtools::install_github (rfrelat/cuspra). A Shiny App was also developed to allow other researchers or stakeholders to easily try the method with their data or simulated data (https://rfrelat.shinyapps.io/CUSPRA). Supplementary material is available online [78].
